# Cryptic species *Hydatigera kamiyai* and other taeniid metacestodes in the populations of small mammals in Serbia

**DOI:** 10.1186/s13071-023-05879-x

**Published:** 2023-07-25

**Authors:** Milan Miljević, Marija Rajičić, Gérald Umhang, Branka Bajić, Olivera Bjelić Čabrilo, Ivana Budinski, Jelena Blagojević

**Affiliations:** 1 Department of Genetic Research, Institute for Biological Research “Siniša Stanković”—National Institute of the Republic of Serbia, University of Belgrade, Bulevar Despota Stefana 142, 11000 Belgrade, Serbia; 2 Nancy Laboratory for Rabies and Wildlife, National Reference Laboratory for Echinococcus Spp, ANSES, Technopôle Agricole Et Vétérinaire, 40009, 54220 Malzéville, CS France; 3 Faculty of Sciences, Department of Biology and Ecology, University of Novi Sad, Trg Dositeja Obradovića 2, 21000 Novi Sad, Serbia

**Keywords:** Parasite, *Taenia*, Rodents, *cox1*, 12*S* rDNA, Phylogenetics

## Abstract

**Background:**

*Hydatigera* (Cestoda: Taeniidae) is a recently resurrected genus with the description of a new species, *Hydatigera kamiyai*, a cryptic entity within the *Hydatigera taeniaeformis* species complex. Rodents are intermediate hosts and correct taxonomic identification of *H. taeniaeformis* sensu lato (s.l.) species is difficult without the use of molecular methods. The aim of this study was to identify and explore the genetic diversity of *Hydatigera* and other taeniid species.

**Methods:**

Ten different small mammals species (856 individuals) (*Rattus rattus*, three *Apodemus*, three Arvicolinae and three Soricidae species) were examined from 2013 to 2023. Captured animals were visually examined for cysts and visible lesions. Two markers were used for amplification and sequencing: *cox1* and 12*S* rDNA.

**Results:**

Molecular analysis of cysts and visible lesions revealed four taeniid species: *Hydatigera kamiyai*, *H. taeniaeformis* sensu stricto (s.s.), *Taenia martis* and *T. crassiceps*. *Hydatigera kamiyai* was found in *Apodemus flavicollis*, *A. agrarius*, *Microtus arvalis* and *Crocidrua leucodon*, while *H. taeniaeformis* s.s. is registered in *R. rattus*. *Hydatigera kamiyai cox1* sequences clustered with European populations and showed at least 25 nucleotid differences compared to Asian, African, Australian and one of our isolates of *H. taeniaeformis* s.s acquired from a rat, followed by large sequence distances (9.4% to 12.9%), indicating clear molecular distinction of two species.

**Conclusions:**

This is one of the few mitochondrial gene-based studies performed after the description of cryptic entities within the *Hydatigera taeniaeformis* s.l. complex and represents a valuable contribution to understanding of genetic diversity, host suitability and geographic distribution of these tapeworm species. Also, our study provides an important basis of molecular data from this part of Europe for further studies. We emphasize the importance of additional studies of intermediate hosts, especially rats from Europe and *Apodemus* spp. and voles from Asia and Africa.

**Graphical abstract:**

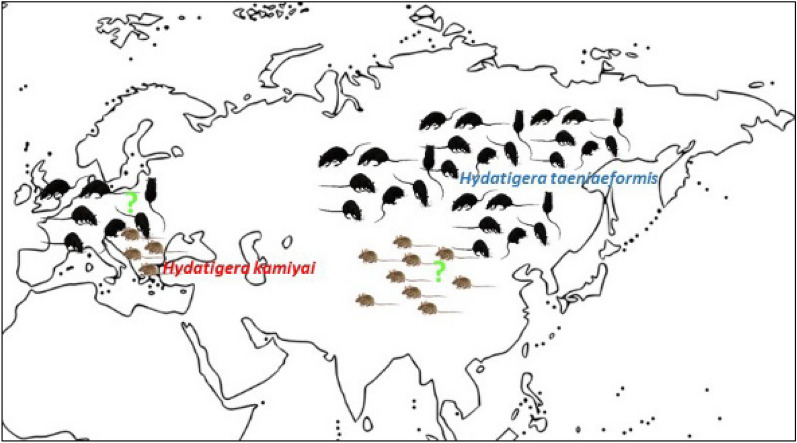

**Supplementary Information:**

The online version contains supplementary material available at 10.1186/s13071-023-05879-x.

## Background

The cyclophyllidean family, Taeniidae, consists of four genera: *Taenia* Linnaeus, 1758; *Echinococcus* Rudolphi, 1801, *Hydatigera* Lamarck, 1816; and *Versteria*. Until recently, only two genera were recognized. Nakao et al. [1] proposed the creation of a new genus, *Versteria*, for *Taenia mustelae* and the resurrection of *Hydatigera* for *Taenia taeniaeformis, Taenia parva* and *Taenia krepkogorski*. There have also been other recent changes, with the description of a new species, *Hydatigera kamiyai*, a cryptic entity within the *Hydatigera taeniaeformis* species complex. Finally, *Hydatigera* genus consists of four valid species: *H*. *kamiyai*, *H. taeniaeformis* s.s., *H. krepkogorski* and *H. parva* [1, 2]. *Hydatigera kamiyai* is distributed across Europe to western Siberia, with an unexplained isolated case in Japan, while *H. taeniaeformis* s.s. probably originated in Asia but has spread worldwide [2]. In addition, there is a third cryptic lineage within the *H. taeniaeformis* complex that is restricted to the Mediterranean region, but its taxonomic status is still unclear, and it is currently referred to as *Hydatigera* sp. [2, 3].

The life cycle of taeniid cestodes includes two obligate mammalian hosts. Carnivorous or omnivorous animals are definitive hosts, while other mammals, particularly rodents, play an important role as intermediate hosts. Some species have zoonotic potential due to human infection by the larval stage [4]. In addition to detecting infection in the final host, knowledge of the role of rodents in the parasite life cycle and identification of parasite species is also important for determining potential transmission and management of zoonoses [5, 6]. Several species of Arvicolinae are considered the most important intermediate host for *Echinococcus multilocularis* [5, 7], a parasite that causes one of the most severe parasitic infections in humans. Infection with *H. taeniaeformis* s.l. is widespread in wild rodents (rats, voles and mice) and cats as the main definitive host [2, 3, 8]. Cysticercosis of *H. taeniaeformis, Taenia crassiceps*, *T. martis* and *Versteria sp*. develops naturally in rodents and accidentally in humans. The definitive hosts for *T. crassiceps* are various Carnivora species, while the carriers of *T. martis* and *Versteria* sp. are mainly mustelids [4]. In some tapeworm genera, differences between species (in adults and larvae stage) are subtle because of high morphological plasticity. Identification could be difficult without the use of molecular methods to ensure correct taxonomic identification, especially in the not yet fully developed larval stage [7, 9–12]. *Hydatigera taeniaeformis* was recently confirmed as a complex of three cryptic entities that differ very little morphologically (practically only in the dimensions of the rostellar hooks) despite extraordinary genetic divergence [2], highlighting the need of molecular identification. Since then, several studies have been conducted on the *H. taeniaeformis* s.l. complex, mainly based on analyses of the mitochondrial marker cytochrome c oxidase subunit 1 (*cox1*) [8, 13–18]. However, due to the small number of studies, geographic distribution and host susceptibility of this group of parasites remains poorly undertood. In this context, aims of the present study were: (i) to identify presence of *Hydatigera* and other taeniid species in various small mammals using molecular methods; (ii) to investigate genetic diversity and provide molecular data for further studies on intraspecific variation within the *H. taeniaeformis* s.l. complex. Considering the geographical location of the studied area and the targeted intermediate hosts, we expected the presence of *H. kamiyai* in our sample. Moreover, this is the first molecular and genetic study of larval taeniid infections in small mammals in Serbia.

## Methods

### Trapping of small mammals and necropsy

A total of 856 small mammals from 45 different locations (Additional file 1: Figure S1) in Serbia were captured from 2013 to 2023 (March-October). Ten different species of small mammals were identified morphologicaly: *Apodemus flavicollis* (*n* = 520), *A. agrarius* (*n* = 152), *A. sylvaticus* (*n* = 34), *Microtus arvalis* (*n* = 48), *M. subterraneus* (*n* = 17), *Myodes glareolus* (*n* = 52), *Rattus rattus* (n = 10), *Crocidura suaveolens* (*n* = 13), *C. leucodon* (*n *= 7) and *Sorex araneus* (*n* = 3). The animals were trapped using Longworth live traps containing dry hay and wheat grains in the nest box and baited with a mixture of oatmeal and sardines. Traps set in the afternoon were checked in the early morning, and captured animals were transported to the laboratory in suitable cages. Captured animals were killed and visually examined during necropsy for cysts and visible lesions in organs, abdominal and thoracic cavities. All tissue lesions were stored at −20 ℃ for further molecular studies.

### DNA extraction, amplification and sequencing

Genomic DNA from parasite specimens (small pieces of host’s infected tissue) was extracted using the Quick-DNA MicroPrep Kit (Zymo Research, USA) according to the manufacturer's instructions, preceded by an overnight digestion with proteinase K. Two markers were used for amplification and sequencing: a fragment of approximately 400 bp of the cytochrome c oxidase subunit 1 (*cox1*) was amplified using the primers JB3 and JB45 [19] and a fragment of approximately 350 bp of mitochondrial (mt) 12S rDNA was amplified using the primers P60 for and P375 rev [20]). Primer sequences and PCR details and conditions are given in Additional file 1: Tables S1 and S2. The amplification products were separated by agarose gel electrophoresis, stained with Midori Green Direct (Nippon Genetics Europe), visualised on a Bio-Rad Gel Doc 1000 (Bio-Rad Laboratories, Hercules, California, USA) and subsequently sent for commercial sequencing in both directions.

### Data analysis

#### Molecular and phylogenetic analyses

Phylogenetic analyses were conducted separately for each molecular marker. Sequences were aligned and visually inspected using Clustal W in MEGA (v.11) software. Sequences were trimmed to a uniform length of 318 nucleotides for *cox1* gene and 238 nucleotides for 12S rDNA and compared with sequences deposited in the GenBank database. Sequences deposited from 2010 to the present were used to correspond to the period of collection of our samples. A Neighbour-joining tree was constructed with MEGA (v.11) using the Tamura-Nei model, with 10.000 bootstrap replicates.

### Population genetic analysis

Species’ identity was established by matching the obtained sequences with ones in GenBank using the BLAST tool. The genetic diversity (number of haplotypes, haplotype diversity and nucleotide diversity) and neutrality indices (Fu’s Fs and Tajima’s D) were calculated using DnaSP 6.12.03 [21]. A median-joining network [22] was constructed with 13 *cox1* nucleotide sequences from Serbia (present study) and 28 from the GenBank database, using PopART 1.7. Pairwise nucleotide sequence divergences were calculated using the Kimura 2-parameter (K2P) model [23] with a gamma setting of 0.5 in MEGA (V.11) software.

## Results

Visible lesions and cysts were detected in 57 animals (6.7%). The cysticercus form of the parasite was predominantly found in the liver (75.4%), making it the most common site of infestation. Following the liver, the thoracic cavity accounted for the next highest occurrence (19.3%). Additionally, a smaller proportion of cases involved the mesentery and abdominal wall (5.3%). All observed pathological changes exhibited the characteristic morphology of classical metacestodes, representing the typical form of cysticercus. However, one exceptional case was identified, characterized by a unique phenomenon known as the budding of cysticerci. This rare occurrence resulted in a significant and distinctive infection (Additional file 1: Figure S2).

Of the total visible lesions and cysts observed in 57 animals, successful amplification of cox1 and 12S rDNA fragments was achieved in 16 and 13 larval samples, respectively. Amplification of 12*S* rDNA gene was performed only when *cox1* was negative. *Hydatigera kamiyai* was found in 17 *A. flavicollis*, one *A. agrarius,* two *M. arvalis* and one *C. leucodon*. *Hydatigera taeniaeformis* s.s. was registred in one rat (*R. rattus*). *Taenia martis* was found in four *A. flavicollis*, one *A. sylvaticus* and one *M. glareolus*, while *T. crassiceps* was detected in only one animal (*M. arvalis*) (Table 1). Neither a cyst nor visible lesions were detected in *M. subterraneus*, *C. suaveolens* *and S. araneus*. All 13 *cox1* sequences and eight 12*S* rDNA sequences of *H. kamiyai* had 99.37–100% identity with NC037071 sequence from GenBank**.** One *cox1* sequence of *H. taeniaeformis* s.s had 100% identity with KT693060 and MH938573 previously published sequences. Two larval samples were identified as *T. martis*, based on 100% identity of *cox1* marker with isolated origin from a human brain from France (KP198618). Four 12*S* rDNA sequences of *T. martis* had 100% identity with three sequences from the GenBank database (LT837855, KT943415, AB731758). Comparison between our 12S rDNA sequence of *T. crassiceps* and the sequences from the GenBank database (AF216699 and MN505206) showed 100% identity.

**Table 1:** Host data and GenBank accession number for all sequences

Genus	ID	Host species, gender	Localities, year	Parasite species	GenBank ID	
					12S rDNA	COX1
*Rattus*	3/23	*R. rattus,* f	Belgrade-New Belgrade, 2023	*H.taeniaeformis* s.s	/	OQ832778
*Apodemus*	3669	*A. flavicollis*, m	Belgrade-Košutnjak, 2013	*H. kamiyai*	OQ834423	/
	3754	*A. flavicollis*, f	Petnica 2014	*H. kamiyai*	OQ834422	/
	3922	*A. flavicollis*, m	Petnica, 2014	*H. kamiyai*	OQ834421	/
	4440	*A. flavicollis*, f	Belgrade-Ada Ciganlija, 2021	*H. kamiyai*	OQ834420	/
	4471	*A. flavicollis*, m	Stara planina -Senokos, 2021	*H. kamiyai*	OQ834419	/
	4521	*A. flavicollis*, f	Ada Ciganlija, 2021	*H. kamiyai*	OQ834418	/
	3702	*A. flavicollis*, f	Belgrade-Košutnjak, 2014	*T. martis*	OQ834427	/
	3712	*A. flavicollis*, f	Petnica, 2014	*T. martis*	OQ834428	/
	3853	*A. flavicollis*, f	Misača, 2014	*T. martis*	/	OQ592885
	4155	*A. flavicollis*, f	Petnica, 2017	*T. martis*	/	OQ592884
	3708	*A. flavicollis*, f	Petnica, 2014	*H. kamiyai*	/	OQ569719
	3823	*A. flavicollis*, m	Misača, 2014	*H. kamiyai*	/	OQ569721
	3961	*A. flavicollis*, m	Belgrade-Košutnjak, 2015	*H. kamiyai*	/	OQ569723
	4158	*A. flavicollis*, m	Petnica, 2017	*H. kamiyai*	/	OQ569724
	4587	*A. flavicollis*, f	Belgrade-Autoput, Kvantaš, 2022	*H. kamiyai*	/	OQ569725
	4591	*A. flavicollis*, f	Belgrade-Autoput, Kvantaš, 2022	*H. kamiyai*	/	OQ569726
	3711	*A. flavicollis*, f	Petnica, 2014	*H. kamiyai*	/	OQ569728
	3758	*A. flavicollis*, f	Ruski Krstur, 2014	*H. kamiyai*	/	OQ569729
	3895	*A. flavicollis*, m	Ruski Krstur, 2014	*H. kamiyai*	/	OQ569730
	3954	*A. flavicollis*, m	Belgrade-Košutnjak, 2015	*H. kamiyai*	/	OQ569731
	4627	*A. flavicollis*, m	Belgrade-Autoput, Kvantaš, 2022	*H. kamiyai*	/	OQ569727
	3873	*A. agrarius*, m	Misača, 2014	*H. kamiyai*	/	OQ569722
	4152	*A. sylvaticus* f	Petnica, 2017	*T. martis*	OQ834429	/
*Microtus*	3761	*M. arvalis* f	Ruski Krstur, 2014	*H. kamiyai*	OQ834424	/
	4449	*M. arvalis*, f	Belgrade-Ada ciganlija, 2021	*H. kamiyai*	OQ834425	/
	4514	*M. arvalis*, f	Belgrade -Ada Ciganlija, 2021	*T. crassiceps*	OQ834430	/
*Myodes*	4161	*M. glareolus*, f	Petnica, 2017	*T. martis*	OQ834426	/
*Crocidura*	3989	*C. leucodon*, m	Cer, 2015	*H. kamiyai*	/	OQ569720

*ID* protocol number, *m* male, *f* female

**Fig. 1:**
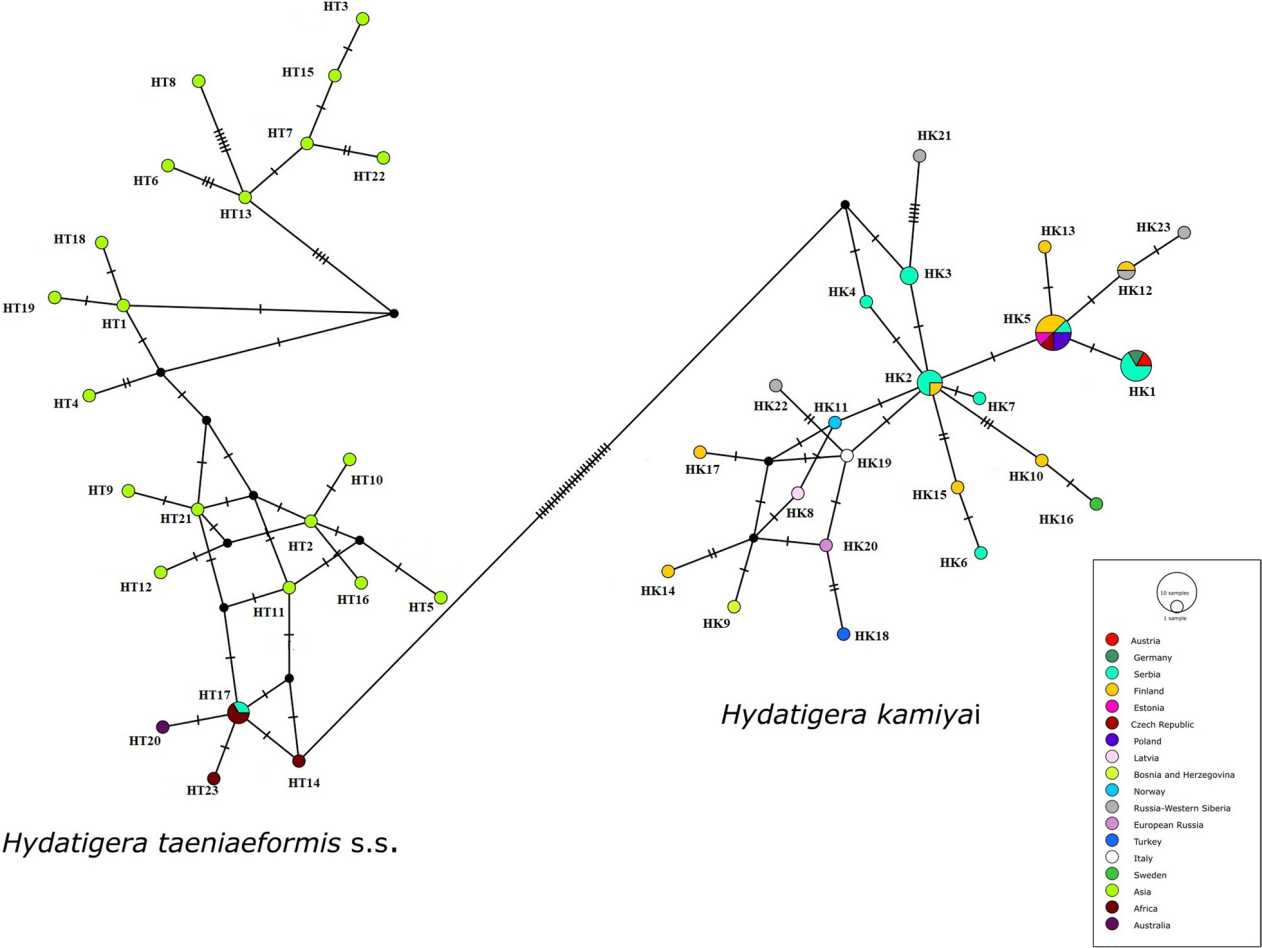
Median-joining network of *Hydatigera kamiyai* isolates from Serbian small mammals and other different hosts from Europe and Western Siberia based on *cox1* gene sequences (as given in Table 2). Haplotype network also shows relations between European and West Siberian *H. kamiyai* isolates and Asian, Australian, African and one of our isolates of *Hydatigera taeniaeformis* s.s. (19 sequences [2]; 1 sequence [27]; 1 sequence [46]; 1 sequence [47]; 1 sequence [18])

In this study, we recorded seven haplotypes of *cox1* gene, out which four were reported for the first time (HK3, HK4, HK6 and HK7) and submitted to GenBank. Thirteen *H. kamiyai cox1* gene partial sequences from this study and 28 from GenBank database corresponded to 23 haplotypes (HK1-HK23), whereas 19 were already known from deposited sequences (Table 2). Lavikainen et al. [2] described 22 haplotypes (named B1-B22 in original study) using 396 bp of the gene that had to be truncated in this study to the 318 bp length, resulting in a reduction of the number of haplotypes to 19. New haplotypes HK4, HK6 and HK7 were represented with a single sequence each, and haplotype HK3 was represented with two sequences. Eight sequences from our study clustered with previously recorded B16 (HK1), B7 (HK2) and B3/B12 (HK5) (Table 2). According to the median-joining network, all the 13 *H. kamiyai cox1* sequences from this study clustered with populations from Europe. Two main haplotypes (HK1 and HK5) were observed, representing 34.1% of the total sample of sequences. A high presence of haplotypes HK1 and HK2 is registered in our samples. HK2 takes a central position in the haplotype network, with no more than 1–6 nucleotide differences from other haplotypes. European haplotypes had at least 25 mutation steps from Asian, African and Australian isolates (*H. taeniaeformis* s.s.). One isolate of *H. taeniaeformis* s.s. from this study derived from rat shared same haplotype with samples from Africa and clustered with African populations (Fig. 1). These results confirm and highlight the differential geographic distribution and molecular distinctiveness in *H. taeniaeformis* s.l. complex.

**Table 2:** Haplotypes (*cox1*) of *Hydatigera kamiyai* found in Serbia and other European countries

*cox 1* haplotypes (HK1-HK23)	*Cox1* haplotypes original name and GenBank ID	Country	Hosts	References
HK1	B16 (JQ663994) JN882301 OQ569719* OQ569720* OQ569721* OQ569729*	Germany Austria Serbia Serbia Serbia Serbia	*Felis silvestris catus,* *Microtus arvalis* *Apodemus flavicollis* *Crocidura leucodon* *Apodemus flavicollis* *Apodemus flavicollis*	[25] [2]
HK2	B7 (KT693082) OQ569722* OQ569723* OQ569726*	Finland Serbia Serbia Serbia	*Felis silvestris catus* *Apodemus agrarius* *Apodemus flavicollis* *Apodemus flavicollis*	[2]
HK3	SRB1 (OQ569724)* SRB1 (OQ569730)*	Serbia Serbia	*Apodemus flavicollis* *Apodemus flavicollis*	
HK4	SRB2 (OQ569725)*	Serbia	*Apodemus flavicollis*	
HK5	B3 (EU861478) B12 (KT693087) AB731761 MT407624 JQ837814 MN514030 MN514032 OQ569727*	Finland Finland Finland Estonia Czech Republic Poland Poland Serbia	*Felis catus,* *Felis silvestris catus* *Felis catus* *Felis catus* *Falco tinnunculus* *Microtus arvalis* *Myodes glareolus* *Apodemus flavicollis*	[15] [2]
HK6	SRB3 (OQ569728)*	Serbia	*Apodemus flavicollis*	
HK7	SRB4 (OQ569731)*	Serbia	*Apodemus flavicollis*	
HK8	B1 (KT693076)	Latvia	*Apodemus flavicollis*	[2]
HK9	B2 (KT693077)	Bosnia and Herzegovina	*Apodemus flavicollis*	[2]
HK10	B4 (KT693079)	Finland	*Microtus agrestis*	[2]
HK11	B5 (KT693080)	Norway	*Microtus agrestis*	[2]
HK12	B6 (KT693081) B19 (KT693091)	Finland, Russia-Western Siberia	*Felis silvestris catus* *Microtus agrestis*	[2]
HK13	B8 (KT693083)	Finland	*Felis silvestris catus*	[2]
HK14	B9 (KT693084)	Finland	*Felis silvestris catus*	[2]
HK15	B10 (KT693085)	Finland	*Felis silvestris catus*	[2]
HK16	B11 (KT693086)	Sweden	*Arvicola amphibius*	[2]
HK17	B13 (KT693088)	Finland	*Felis silvestris catus*	[2]
HK18	B14 (EU544596)	Turkey	*Apodemus sylvaticus*	[2]
HK19	B15 (FN547850)	Italy	*Felis silvestris silvestris*	[2]
HK20	B17(KT693089) B18 (KT693090)	European Russia European Russia	*Apodemus uralensis* *Apodemus uralensis*	[2]
HK21	B20 (KT693092)	Russia-Western Siberia	*Alticola strelzowi*	[2]
HK22	B21 (KT693093)	Russia-Western Siberia	*Mus musculus*	[2]
HK23	B22 (KT693094)	Russia-Western Siberia	*Myodes rutilus*	[2]

Bold-samples/sequences from this study

**Fig. 2:**
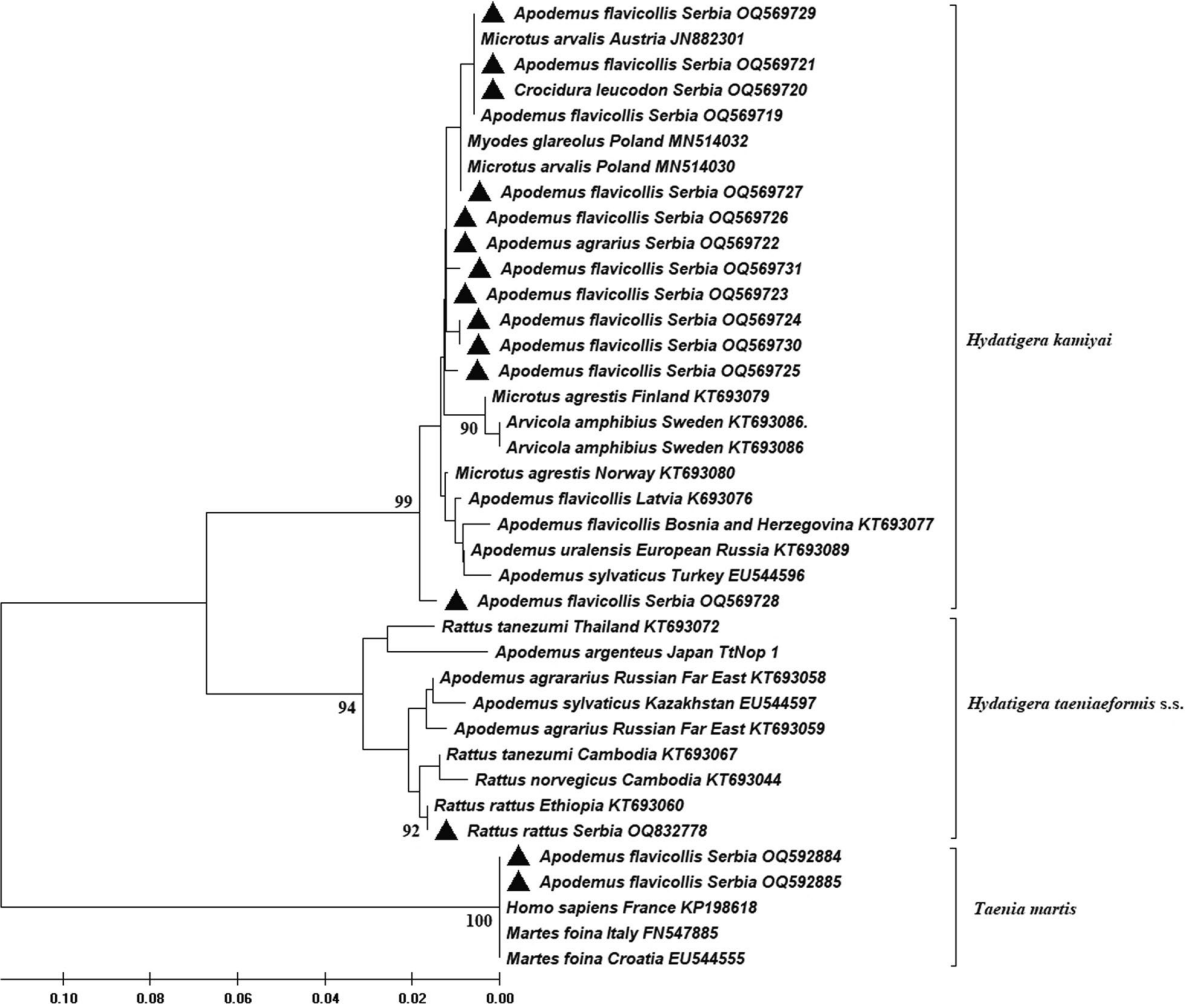
Phylogenetic tree of the *Hydatigera taeniaeformis* species complex based on 318 bp *cox1* gene sequences. The tree was inferred using neighbour-joining method constructed with MEGA (v.11) using the Tamura-Nei model, with 10,000 bootstrap replicates. Values > 90% are shown. The sequences of *Taenia martis* from this and other studies are shown in the tree. Black triangles indicate our isolates

**Fig. 3:**
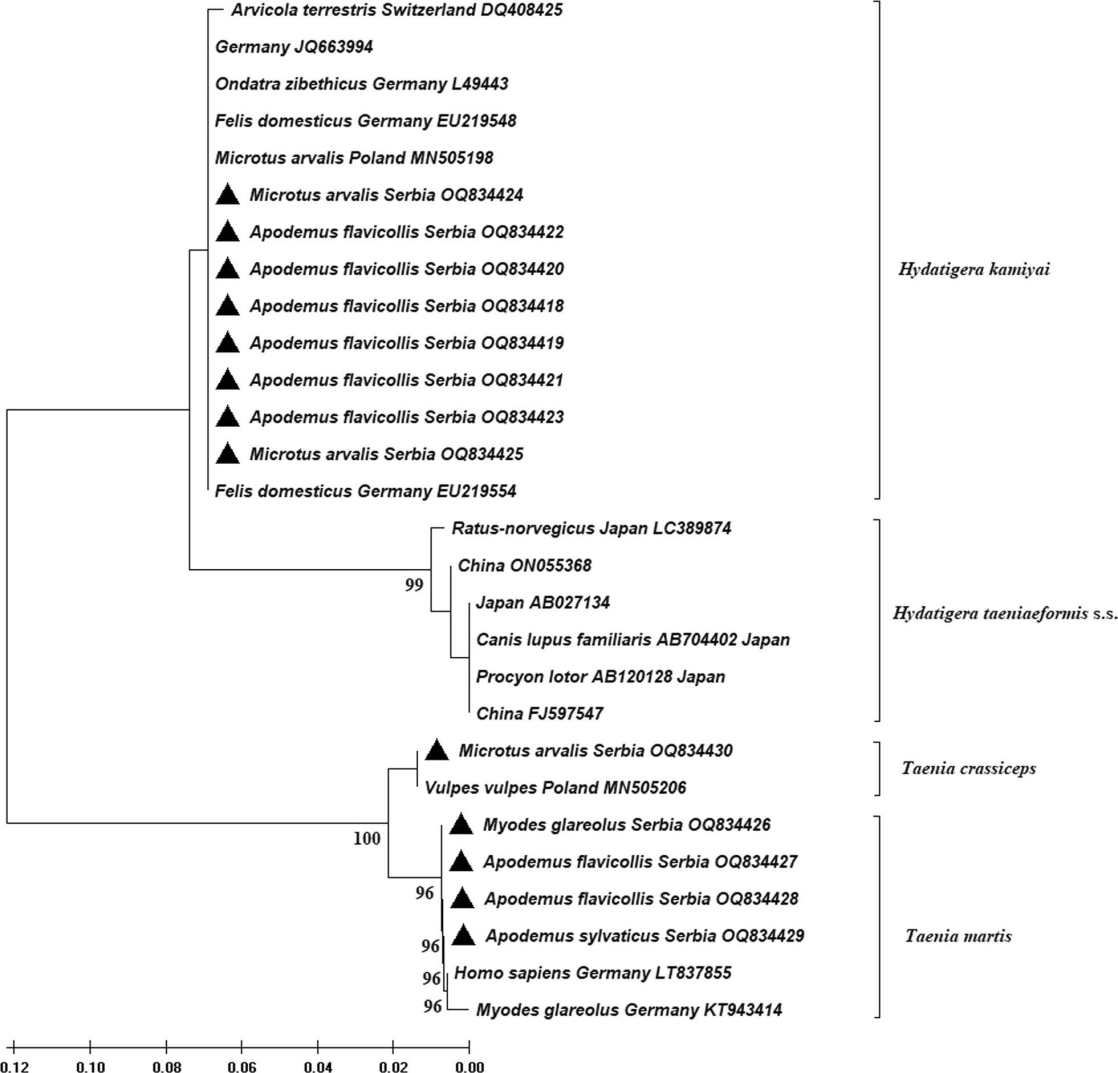
Phylogenetic tree of the *Hydatigera taeniaeformis* species complex based on 238 bp 12*S* rDNA gene sequences. The tree was inferred using neighbour-joining method constructed with MEGA (v.11) using the Tamura-Nei model, with 10,000 bootstrap replicates. Values > 90% are shown. The sequences of *Taenia martis* and *T. crassiceps* from this and other study are shown in the tree. Black triangles indicate our isolates

Sequences of *cox1* gene from this study differed by 0–2.7% from *H. kamiyai* isolates from Europe and western Siberia. On the other hand, pairwise divergences with Asian, African, Australian and a single rat-derived isolate of *H. taeniaeformis* s.s from Serbia were high, ranging from 9.4 to 12.9%. In addition, 12S rDNA sequences from this study differed up to 0.42% from the rest of European isolates and by 7.46–8.07% from Asian isolates. In the phylogenetic analyses results the Serbian samples were always clustered with the European ones and were distant from the Asian and African isolates (Figs. 2, 3).

Polymorphism analysis of 13 *H. kamiyai cox1* sequences showed eight polymorphic sites, three parsimony informative sites and five singleton variable sites, resulting in seven haplotypes. Haplotype diverstiy (Hd) and nucleotide diversity (π) of 0.872 and 0.00637, respectively, were observed, with no significant negative Tajima’s *D* (0.82982) and Fu’s F (−2.255) values (Table 3). All 12S rDNA sequences (238 bp) of *H. kamiyai* from this study corresponded to the same haplotype, revealing no genetic diversity.

**Table 3:** Summary statistics of DNA polymorphism and neutrality tests based on sequences of the *cox1* gene from this study

Indices	*Cox1* (318 bp)
No. of sequences	13
Variable (polymorphic) sites	8
Parsimony informative sites	3
Singleton variable site	5
No. of haplotypes	7
Haplotype diversity (Hd)	0.872
Nucleotide diversity (π)	0.00637
Nucleotide differences (k)*	2.026
Tajima’s *D*	-0.82982 (NS)
Fu's Fs	−2.255 (NS)

*Average number of pairwise nucleotide differences (k)

NS = not significant

## Discussion

In the present study, ten different species of small mammals (*R. ratus*, three *Apodemus*, three Arvicolinae and three Soricidae species) were examined for the presence of taeniid metacestodes. Despite targeting intermediate host, *E. multilocullaris* was not recorded. This can be explained by the absence of samples collected in a hot-spot area of alveolar echinococcosis in the northern part of Serbia (Vojvodina, Srem) [11, 24]. This result may also indicate that the disease is still present only in the registered focal area and has not spread south of the Sava and Danube Rivers. Molecular analysis of cysts and visible lesions revealed four taeniid species: *H. kamiyai*, *H. taeniaeformis* s.s., *T*. *martis* and *T. crassiceps* in voles, rats and *Apodemus* spp. These wild rodents are suitable intermediate hosts for registered parasite species, which is consistent with other studies [2, 7, 15, 25, 26]. Shrews (family Soricidae) are not considered intermediate hosts for *Hydatigera* species, but we found *H. kamiyai* at larval stage in one *C. leucodon*. To the best of our knowledge, this is the first record of *H. kamiyai* in a small insectivorous mammal. Similarly, Al-Sabi et al. [6] found *H. taeniaeformis* s.s. infection in only one *S. araneus* in Denmark and noted that this does not necessarily mean that cysts can successfully develop in these hosts, making them a reservoir for the parasite, and implicating their exposure to infectious eggs. We did not recorded any cysts or lesions in *Sorex* shrews of our sample. Catalano et al. [13] did not detect *H. taeniaeformis* s.s. in 28 shrews (*Crocidura*) examined in Senegal.

Of the other animals infected with *H. kamiyai* in our study, 18 belong to *Apodemus* (predominantly *A. flavicollis*) and two belong to *M. arvalis* (Arvicolinae). One *R. rattus* from our study was infected with *H. taeniaeformis* s.s. These results are in accordance with the fact that *H. kamiyai* mainly uses Arvicolinae and mice of the genus *Apodemus* as intermediate hosts, whereas *H. taeniaeformis* s.s. is restricted to murine rodents (mainly rats) [2]. The presence of *H. kamiyai* was also recently confirmed by molecular analyses in the same group of intermediate hosts in Europe such as *M. arvalis* and *M. glareolus* in Poland [15] and *Ondatra zibethicus* in Luxembourg [8]. Considering that we conducted the study on a large sample and a wide range of intermediate host species from Serbia (Southeastern Europe) and found *H. kamiyai* in members of Arvicolinae and *Apodemus* spp., our results are concordant with the current biogeography and host range of this recently described cryptic species and contribute to its clarification. On the other hand, it should be noted that we provided the sequence of *H. taeniaeformis* s.s from the black rat, but molecular studies of *Hydatigera taeniaeformis* s.l. in Europe are limited to Arvicolinae and *Apodemus* hosts [2, 8, 15]. Similarly, molecular studies of *Hydatigera taeniaeformis* s.l. from areas in Asia and Africa have been conducted mainly on intermediate hosts from the family Muridae [2, 13, 16, 17] and several individuals of *Apodemus* spp. that were found to be infected with *H. taeniaeformis* s.s. [2, 27], a parasite species for which members of the family Muridae (mainly rats) are considered suitable hosts. The previous discovery of *H. taeniaeformis* s.s. in the genus *Apodemus* in Asia (Fig. 2) and the lack of molecular studies of *Hydatigera taeniaeformis* s.l. in rats in Europe impose the need for additional research to obtain a more accurate view of the intermediate host spectrum from the different species of the genus. In a molecular study from Brazil, *H. taeniaeformis* s.s. was detected for the first time in the non-murine rodent Ingram's squirrel (*Guerlinguetus ingrami*), and the authors also point out that the purported host specificity of cryptic *Hydatigera* species is not strict [14]. Further testing on different host species from different areas is needed (especially rats from Europe and Arvicoline and *Apodemus* from Asia), and the possibility of infection of other small mammal species, e.g., shrews, should be considered. According to the median-joining haplotype network in the present study, *H. kamiyai* haplotypes from this study were clustered with other haplotypes from Europe and had at least 25 mutation steps from Asian, African and Australian haplotypes of *H. taeniaeformis* s.s., indicating clear molecular distinction of two species. Alvi et al. [17] also detected 20 mutation steps from the Pakistani *H. taeniaeformis* s.s. sequences compared to the European isolates (*H. kamiyai*). Pairwise divergences of our samples (*H. kamiyai*) with Asian, African and Australian isolates (*H. taeniaeformis* s.s.) from GenBank were high, ranging from 9.4% to 12.9%, supporting existence of two distinct taxa. These results are supported by the variation in the partial sequence of the mitochondrial *cox1* gene between two clades of *H. taeniaeformis* s.l., reaching up to 13.3% [2]. The metacestode in Ingram's squirrel (*G. ingrami*) from Brazil found by Mello et al. [14] was identified as a *H. taeniaeformis* s.s and differed from *H. kamiyai* by 11.3–11.8%. A comparison of the complete mitochondrial (mt) genome between some European and Asian *H. taeniaeformis* s.l. species performed in two studies revealed a difference of 11.8–12.1%, and the presence of two distinct clades referred to as sister taxa, leading to the conclusion that *H. taeniaeformis* represents a species complex [18, 28]. All this recent molecular research and our results support the earlier discovery from the 1990s, when scientists noticed the existence of differences based on four rare laboratory isolates (KRN from the gray rat *Rattus norvegicus* from Malaysia; ACR from the gray red-backed vole *Myodes rufocanus* from Japan; SRN from the gray rat *R. norvegicus* from Japan; BMM from the house mouse *Mus musculus* from Belgium) and several wild isolates of *H. taeniaeformis* [Japan: TtMar, TtTom, TtKaRN (*R. norvegicus*); TtKaAA, TtNop (*A. argenteus*) and China: TtChi (*M. musculus*)]. They noted that the ACR isolate (now known as *H. kamiyai*) differed from the others in terms of infectivity, morphology and genetics (intraspecific variation of isoenzymes, nucleotide variations in the *cox1* gene and protein composition of metacestodes) and concluded that it might be a different strain of *H. taeniaeformis* or even a new species [27, 29–31]. Based on *cox 1* sequence analysis of the 13 *H. kamiyai* isolates, we found 7 haplotypes (Hd: 0.872), which is similar to the diversity level of the *H. kamiyai* specimens from Luxembourg based on 26 *cox 1* sequences and 9 haplotypes defined, with Hd = 0.883 [8]. The authors suggested that *H. kamiyai* in Europe has a heterogeneous genetic structure and the different haplotypes are widely distributed across the continent, which could indicate a long and undisturbed presence of this ubiquitous parasite. The haplotype diversity of *H. taeniaeformis* s.s. from Pakistan composed of 38 *cox1* sequences was 0.757, with 10 haplotypes [17], while the greatest diversity was found in Chinese isolates, where 10 haplotypes were recorded from 13 *cox1* sequences [16].

In this study, two additional parasite species were also detected. *Taenia martis* was identified in *A. flavicollis*, *A. sylvaticus* and *M. glareouls* using both 12S rDNA and *cox1* gene, and these are the first obtained sequences of this tapeworm from Serbia, providing new insights into its genetic diversity. Due to small sample size, of only six sequences in total (for both genes), no relevant population genetic analyses could be performed. Based on morphological identification, *T. martis* was found to infect 4.1% of 588 examined bank voles on Fruška gora mountain in Serbia [32]. Cysticercosis of *T. martis* in rodents appears to occur throughout Europe with variable prevalence, while adult forms infect wild carnivores, mainly martens [4]. Cases of the larval stage have also been detected in humans [33–36] and non-human primates [37, 38]. In 10 years of our research and 856 examined animals, only one small mammal (*M. arvalis*) captured in 2021 was found to be infected with *T. crassiceps*. A low prevalence (0.22%–2.9%) of metacestodes in rodent hosts has also been found in other European countries [5, 7, 39]. In several studies that included definitive and intermediate hosts from Europe, the presence of *T. crassiceps* was not recorded [24, 40–42]. Although this infection is rare, it can be very severe and lead to death or serious pathological changes in intermediate and paratenic hosts [4]. This massive and specific-looking infection, which affected the subcutaneous and pleural cavities in our sample (Additional file 1: Figure S2), was detected and confirmed by PCR analyses (12*S* rDNA gene). In natural intermediate hosts such as rodents, *T. crassiceps* metacestodes reproduce by asexual reproduction, particularly by exogenous budding, which is unique to this species and results in the formation of multiple infective scolices [43–45]. A number of well-documented cases of cysticercosis in humans have been published, most of them from Central Europe (Switzerland, France and Germany) [4]. It appears that this species has serious zoonotic potential and should be monitored in animals in the future.

## Conclusions

Further studies are needed to better understand the specificity of two *Hydatigera* species (*H. kamiyai* and *H. taeniaeformis* s.s.) towards the intermediate host and their geographic distribution, particularly in rats from Europe and *Apodemus* spp. and voles from Asia and Africa. The possibility of infection of other small mammalian species, e.g., shrews, should also be considered. This is one of the few mitochondrial gene-based studies performed after the description of cryptic entities within the *H. taeniaeformis* s.l. complex and represents a valuable contribution to understanding of genetic diversity, host suitability and geographic distribution of these tapeworm species. Also, our study provides an important basis of molecular data from this part of Europe for further studies.

## Supplementary Information

**Additional file 1: Table S1.** Primer sequences used for PCR analysis; **Table S2.** PCR details and conditions for two molecular markers used in this study; **Fig. S1.** Map of sampling sites in Serbia. The circles on the map show places where small mammals were collected. The red circles indicate the places where the animals were infected with some of the taeniids larval stages; **Fig. S2.** Cysticercosis caused by larval *Taenia crassiceps* tapeworm in common vole (*Microtus arvalis*).

## Data Availability

Nucleotide sequences of *cox1* and 12*S* rDNA genes from the present study have been deposited in the GenBank database under the accession numbers OQ569719-OQ569731; OQ834418-OQ834430; OQ832778.
