# Supplementary material for: Cryptic species Hydatigera kamiyai and other taeniid metacestodes in the populations of small mammals in Serbia
Source: Parasit Vectors. 2023 Jul 25;16:250. doi: 10.1186/s13071-023-05879-x (PMC10369706; doi:10.1186/s13071-023-05879-x)
Supplement: Supplementary file 1 — Additional file 1: Table S1. Primer sequences used for PCR analysis; Table S2. PCR details and conditions for two molecular markers used in this study; Fig. S1. Map of sampling sites in Serbia. The circles on the map show places where small mammals were collected. The red circles indicate the places where the animals were infected with some of the taeniids larval stages; Fig. S2. Cysticercosis caused by larval Taenia crassiceps tapeworm in common vole (Microtus arvalis). [file 13071_2023_5879_MOESM1_ESM.docx]

| **Additional file 1: Table S1**. Primer sequences used for PCR analysis. | | |
| --- | --- | --- |
| Gene | Primers | References |
| *Cox1* | JB3 5′-TTT TTT GGG CAT CCT GAG GTT TAT-3′  JB45 5′-TAA AGAAAG AAC ATA ATG AAA ATG-3′ | Bowles et al., 1992 |
| 12*S* rDNA | P60for 5′-TTA AGA TAT ATG TGG TAC AGG ATT AGA TAC CC-3′  P375rev 5′-AAC CGA GGG TGACGG GCG GTG TGT ACC-3′ | von Nickisch-Rosenegk et al., 1999 |

| **Additional file 1: Table S2**.**.** PCR details and conditions for two molecular markers used in the study. | |
| --- | --- |
| ***cox1*** | **12*S* rDNA** |
| 5 μl (10×PCR Dream Taq buffer)  2.5 μl dNTPs (10mM),  2.5 μl of each primer (20 μM)  0.2 μl (1 U Dream Taq polymerase) (Thermo Fisher Scientific) | 5 μl (10×PCR Dream Taq buffer),  2 μl dNTPs (10 mM)  2 μl of each primer (20 μM)  0.2 μl (1 U Dream Taq polymerase) (Thermo Fisher Scientific) |
| initial denaturation at 94 °C for 2 min; followed by 35 cycles of 30 s at 94 °C, 30 s at 52 °C and 30 s at 72 °C;  final extension at 72 °C for 7 min | initial denaturation at 94 °C for 3 min; followed by 40 cycles of 30 s at 94 °C, 1 min at 56 °C and 45 s at 72 °C;  final extension at 72 °C for 2 min |

***All PCRs were performed in a final reaction of 50 μl.**

**
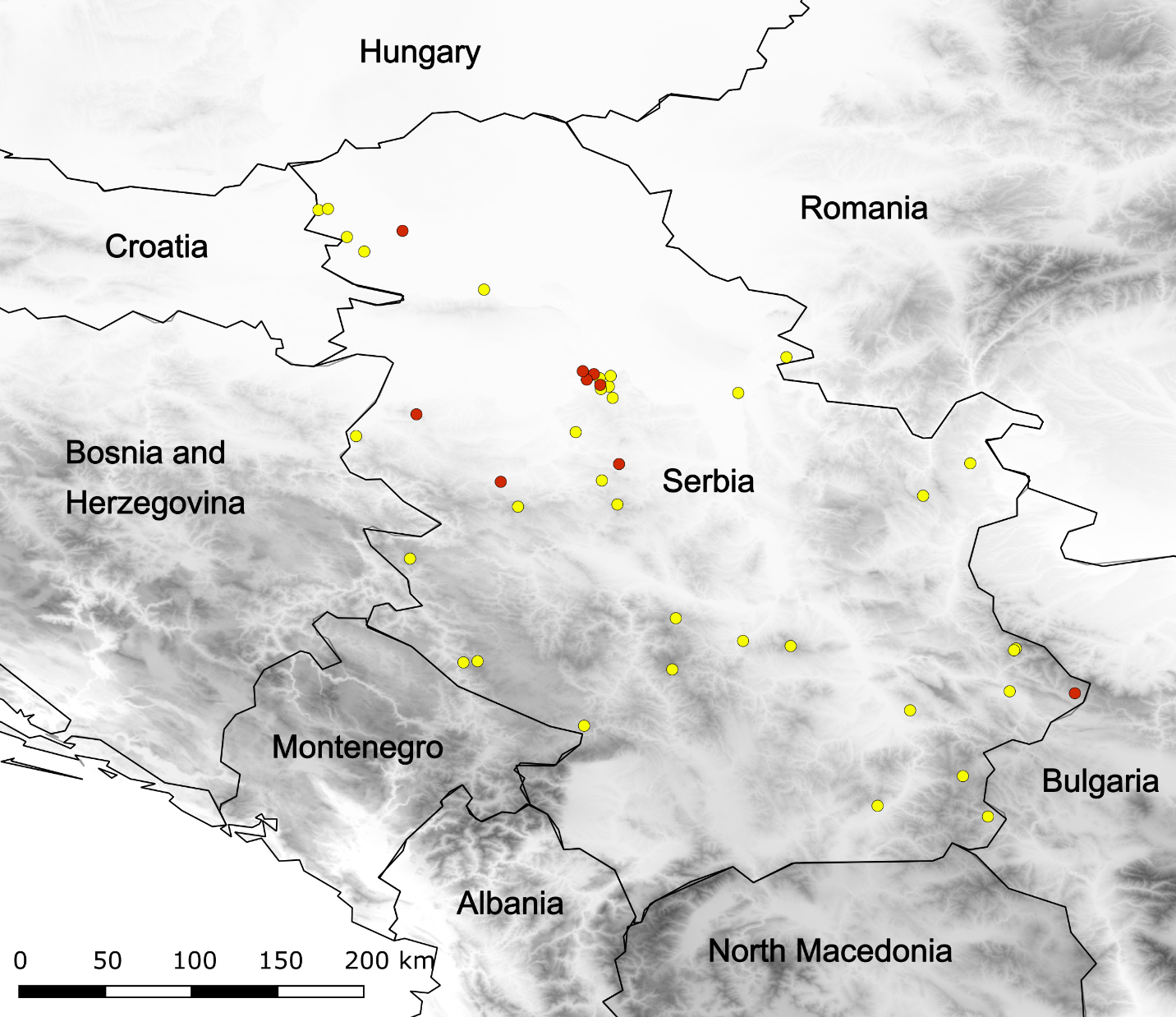
Additional file 1: Fig. S1.** Map of sampling sites in Serbia. The circles on the map show places where small mammals were collected. The red circles indicate the places where the animals were infected with some of the taeniids larval stages.

**
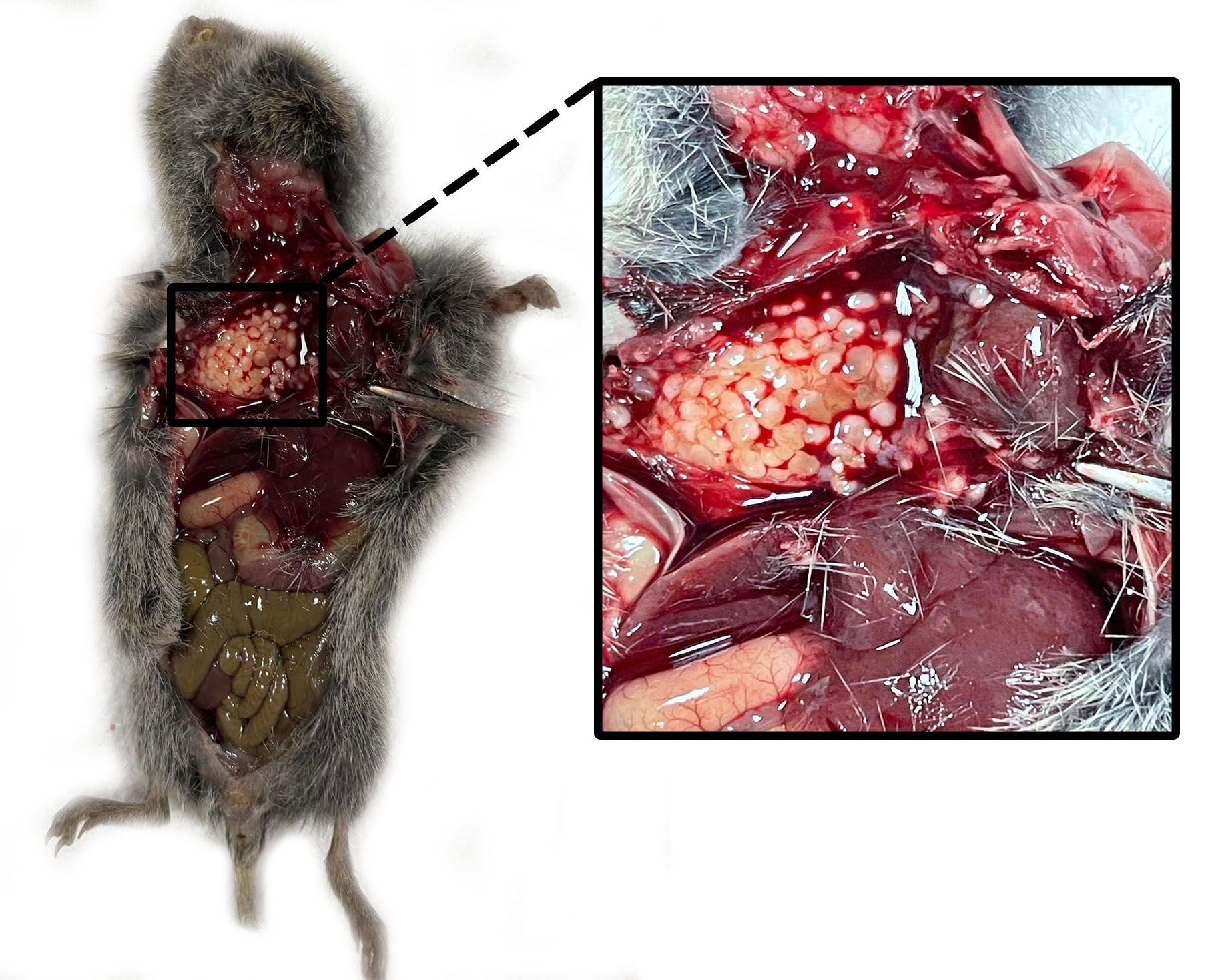
**

**Additional file 1: Fig. S2.** Cysticercosis caused by larval *Taenia crassiceps* tapeworm in common vole (*Microtus arvalis*).
